# Evaluation of the flexion gap with a distal femoral trial component in posterior-stabilized total knee arthroplasty

**DOI:** 10.1186/s43019-022-00142-6

**Published:** 2022-03-10

**Authors:** Goki Kamei, Shigeki Ishibashi, Koki Yoshioka, Satoru Sakurai, Hiroyuki Inoue, Yu Mochizuki, Masakazu Ishikawa, Nobuo Adachi

**Affiliations:** 1 Department of Orthopaedic Surgery, Graduate School of Biomedical and Health Sciences, Hiroshima University, 1-2-3 Kasumi, Minami-ku, Hiroshima, Japan; 2 Department of Orthopaedic Surgery, Hiroshima Prefectural Hospital, 1-5-54, Ujinakanda, Minami-ku, Hiroshima, Japan

**Keywords:** Posterior stabilized, Total knee arthroplasty, Distal femoral trial component, Joint gap size, Joint gap inclination

## Abstract

**Purpose:**

A distal femoral trial component was manufactured, and flexion gap size and inclination were evaluated with or without the distal femoral trial component in total knee arthroplasty (TKA). This study aimed to evaluate the effect of the distal femoral trial component on flexion gap size and joint inclination in posterior-stabilized (PS)-TKA.

**Materials and methods:**

A total of 84 patients with medial osteoarthritis who underwent mobile-bearing PS-TKA using modified gap techniques were included in this retrospective study. The flexion gap size and inclination before and after setting the distal femoral trial component were evaluated and compared with the final gap size and inclination.

**Results:**

The joint gap size and inclination were significantly lower in those with than in those without the distal femoral trial component (*P* = 0.005, *P* < 0.001). The final gap size and inclination were similar to the gap size and inclination with the distal trial component (*P* = 0.468, *P* = 0.158).

**Conclusions:**

The joint gap size and medial tension in PS-TKA were significantly reduced after setting the distal femoral trial component. The flexion gap measured using the distal femoral trial component was similar to that when the final trial component was set. To more accurately perform the gap technique TKA, the flexion gap should be measured using the distal femoral trial component.

## Introduction

Flexion gap in total knee arthroplasty (TKA) is influenced by various factors, including posterior cruciate ligament (PCL) resection, posterior tibial slope, patella height, and extent of distal femoral resection [1–6].

Joint line changes after TKA have an influence on range of motion (ROM), patella height, and mid-flexion laxity [7–9], and thereby TKA has generally been performed by cutting the distal femur, under consideration of the implant thickness, without changing the joint line and patella height. Bone gap was measured after conventional cutting of distal femur and proximal tibia, revealing an increase in flexion gap as compared with that after setting the implant. The distal femoral trial component (Zimmer-Biomet, Warsaw, Indiana, USA) was constructed, and the effect of distal femoral resection on the flexion gap with or without the distal femoral trial component was evaluated in cruciate-retaining (CR) TKA so that there was no influence of posterior cruciate ligament resection [10]. The joint gap size and medial tension were significantly reduced after setting the trial component in CR-TKA. It was assumed that the flexion gap (gap size and inclination) in posterior-stabilized (PS) TKA was affected by the distal femoral trial component as in CR-TKA.

First, this study aimed to evaluate the effect of the distal femoral trial component on the flexion gap size and joint inclination, with or without the distal femoral trial component in PS-TKA. Second, the effect of each measurement (the flexion gap size and inclination with or without the distal femoral trial component) on the final gap size and inclination was examined.

The hypothesis of this study is that the final gap size and inclination are equivalent to gap size and inclination using the distal femoral trial component, rather than gap size and inclination referring to the bone gap (without the distal femoral trial component).

## Methods

### Patients and assessment

**Fig. 1:**
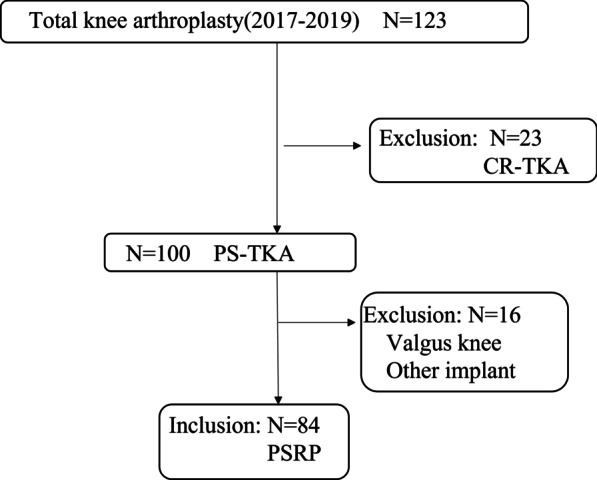
Flow chart of included patients

**Fig. 2:**
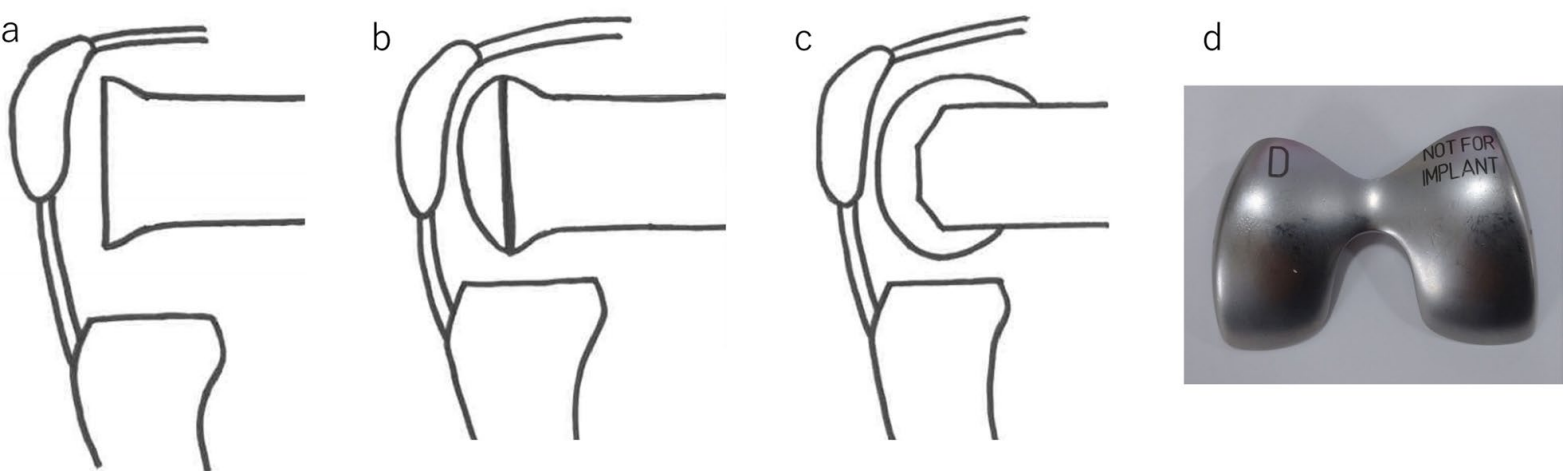
The status of measuring the flexion gap. **a** Before setting the distal femoral trial. **b** After setting the distal femoral trial. **c** After setting the final femoral trial component. **d** Distal femoral trial component

Total knee arthroplasty was performed for 123 patients between May 2017 and March 2019. This retrospective study included 84 patients with medial osteoarthritis who underwent mobile-bearing PS-TKA (Zimmer-Biomet’s PSRP) using the modified gap techniques. Patients included 8 men and 76 women, with a mean age of 77 (range 58–89) years. Demographics and clinical features of all included participants are presented in Table 1. Exclusion criteria were (1) valgus knee: % mechanical axis was more than 50%; (2) cases used other implant. A flow chart of included patients is shown in Fig. 1. Informed consent was obtained from all participants. All procedures were performed by a single surgeon (G.K.) using the modified gap techniques. A medial parapatellar arthrotomy was performed, and the patella did not resurface in all patients. Both anterior and posterior cruciate ligaments were resected. The distal femoral and proximal tibia were cut perpendicular to the mechanical axis. The extension balance was measured using a JDK offset tensor (Stryker, Mahwah, NJ, USA). Soft tissue release was performed until a proper soft tissue balance was obtained; proper soft tissue balance was defined as an intraoperative joint gap inclination of 0° to 3°. The flexion gap size and inclination during 90° flexion were measured using a JDK offset tensor before and after setting the distal femoral trial component, as well as the final femoral trial component after cutting the posterior condyle (Fig. 2) [10, 11]. Referring to the flexion gap size and inclination with and without the distal femoral trial component, the rotational and anteroposterior positions were determined so that the gap size in the final femoral trial component was approximately 2 mm larger than the extension gap size and the inclination in the final trial component was approximately 3°.

**Table 1:** Demographics data and clinical features of all included participants

Patient data	
Age	76.2 (50–88)
Sex (male/female)	7/77
Body mass index (kg/m^2^)	27.1 (17.6–40.7)
Kellgren Lawrence grade (OA grade)	II: 2, III: 19, IV: 63
Range of motion (extension/flexion)	−10.1 (−30 to 0)/111.2 (70–145)
% Mechanical axis (standing view)	1.6 (−66 to 40)

The gap was measured by applying a 30-lb tension after an attempt made to avoid errors from creep elongation by applying a 40-lb tension. The median value was defined as the flexion gap size. When the medial compartment was tight, the gap inclination was positive.

**Fig. 3:**
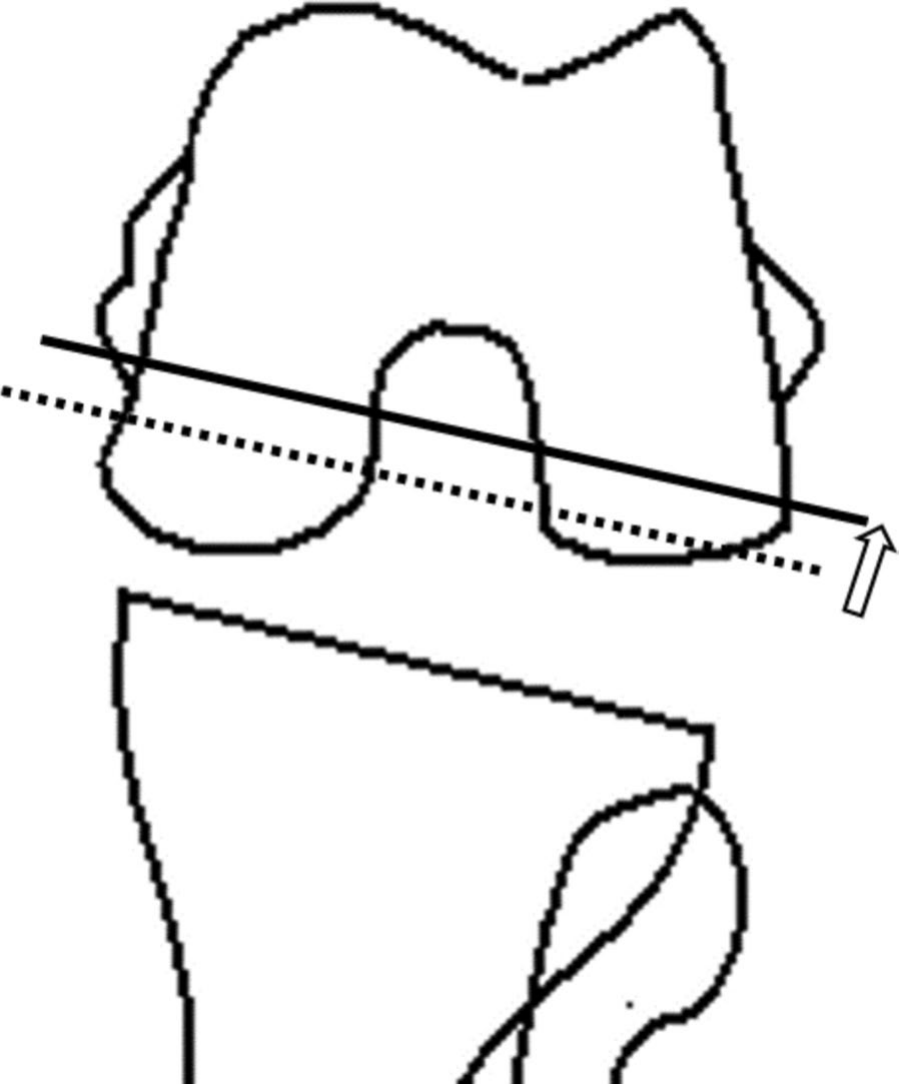
Modified gap technique adjusting the gap size. First, the rotation of the osteotomy line (dotted line) was determined. Dotted line: osteotomy line was determined on the basis of the posterior reference so that the flexion gap is square. Next, the amount of posterior condylar osteotomy was determined. Solid line: actual osteotomy line was determined so that the flexion gap is equal to the extension gap. Anterior translation is positive

**Fig. 4:**
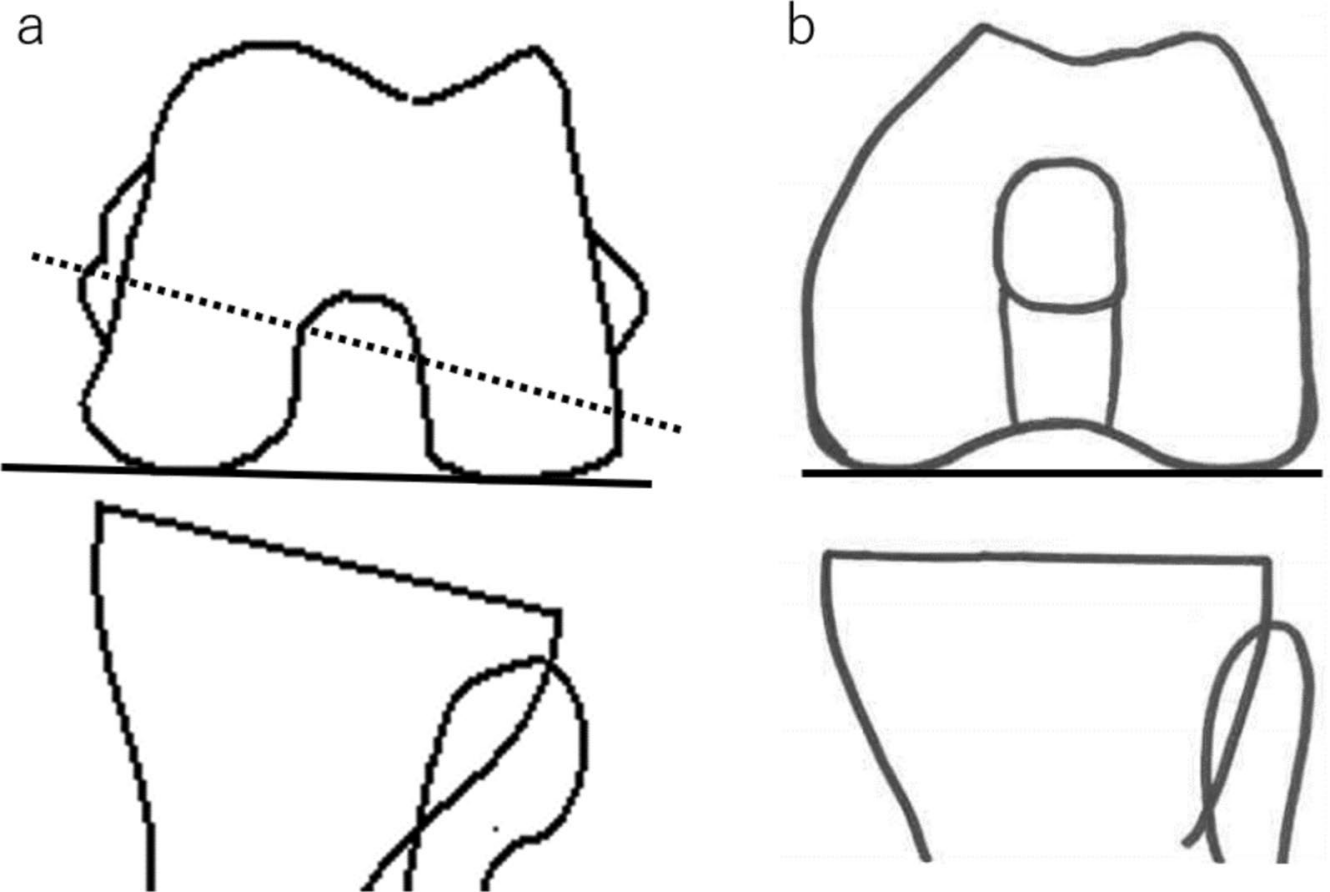
Modified gap technique adjusting the gap inclination. **a** Before posterior condyle osteotomy: The rotation angle of bone resection of posterior condyle is the angle between the line of bone resection (dotted line) and posterior condylar axis (solid line). **b** After posterior condyle osteotomy and setting final femoral trial component: final femoral trial component gap inclination is the angle between posterior condylar axis (solid line) and tibial plateau

The flexion gap size and inclination before and after setting the distal femoral trial component were compared with the value (defined as the final gap size) subtracted by the amount of additional posterior condyle resection (additional posterior condyle resection was defined as the amount of osteotomy from the normal position to the anterior translation, defining the anterior transition as positive) from the final femoral trial component gap size to evaluate whether the gap size before bone resection of the posterior condyle was reflected in the final component gap size (Fig. 3). The flexion gap inclination before and after setting the distal femoral trial component was also compared with the combined value (defined as the final gap inclination) of the final femoral trial component gap inclination and rotation angle of the bone resection in the posterior condyle (defined as positive external rotation osteotomy) to evaluate whether the gap inclination before bone resection of the posterior condyle was reflected in the final component gap inclination (Fig. 4).

The posterior tibial slope angle was defined as the angle between the line perpendicular to the fibular axis and the posterior slope to the tibial plateau. The Blackburne–Peel ratio was used to assess the patellar height. All data were retrospectively collected and analyzed from an institutional database (Hiroshima University Hospital and Hiroshima Prefectural Hospital), and the study was conducted according to the principles of the Declaration of Helsinki.

### Statistical analysis

A power analysis was performed using G*Power to determine the appropriate sample size [12]. Effect size of 0.8, α error probability of 0.05, and power of 0.8 were set for the analysis. A sample size of 15 cases was calculated for paired-*t*-test and 7 cases for Pearson correlation analysis.

Microsoft Excel statistical software was used to analyze the data. Results were expressed as the mean ± standard deviation. Data were compared using paired-*t*-test. Correlations between the flexion gap size and the posterior tibial slope angle and patellar height were assessed using Pearson correlation analysis. *P* < 0.05 was considered statistically significant.

## Results

The mean joint gap size during the 90° flexion was 16.7 ± 0.45 mm without the distal femoral trial component and 16.1 ± 0.46 mm with the distal femoral trial component. The mean gap inclination during 90° flexion was 5.4 ± 0.46° without the distal femoral trial component and 4.3 ± 0.46° with the distal femoral trial component. The mean final gap size and inclination during 90° flexion were 16.1 ± 0.44 mm and 4.6 ± 0.43°, respectively.

The joint gap size and inclination were both significantly lower in those with than those without the distal femoral trial component (*P* = 0.005, *P* < 0.001, respectively; Table 2). The joint gap size and inclination without the distal femoral trial component were both significantly larger than the final gap size and inclination (*P* = 0.033, *P* = 0.016) (Table 3). No significant difference was observed between the gap size with the distal femoral trial component and final gap size (*P* = 0.468), as well as between the gap inclination with the distal femoral trial component and final gap inclination (*P* = 0.158) (Table 4).

**Table 2:** Gap size and inclination without and with the distal femoral trial, and final femoral trial component

	Without distal femoral trial component	With distal femoral trial component	Final femoral trial component
Joint gap size (mm)	16.7 ± 0.45	16.1 ± 0.46	16.1 ± 0.44
Joint gap inclination (°)	5.4 ± 0.46	4.3 ± 0.46	4.6 ± 0.43

**Table 3:** Comparison of gap size and inclination without versus with the distal femoral trial component

	Without distal femoral trial component	With distal femoral trial component	*P* value
Joint gap size (mm)	16.7 ± 0.45	16.1 ± 0.46	0.005
Joint gap inclination (°)	5.4 ± 0.46	4.3 ± 0.46	< 0.001

**Table 4:** Comparison of gap size and inclination without the distal femoral trial component versus with the final femoral trial component

	Without distal femoral trial component	Final femoral trial component	*P* value
Joint gap size (mm)	16.7 ± 0.45	16.1 ± 0.44	0.033
Joint gap inclination (°)	5.4 ± 0.46	4.6 ± 0.4	0.016

**Fig. 5:**
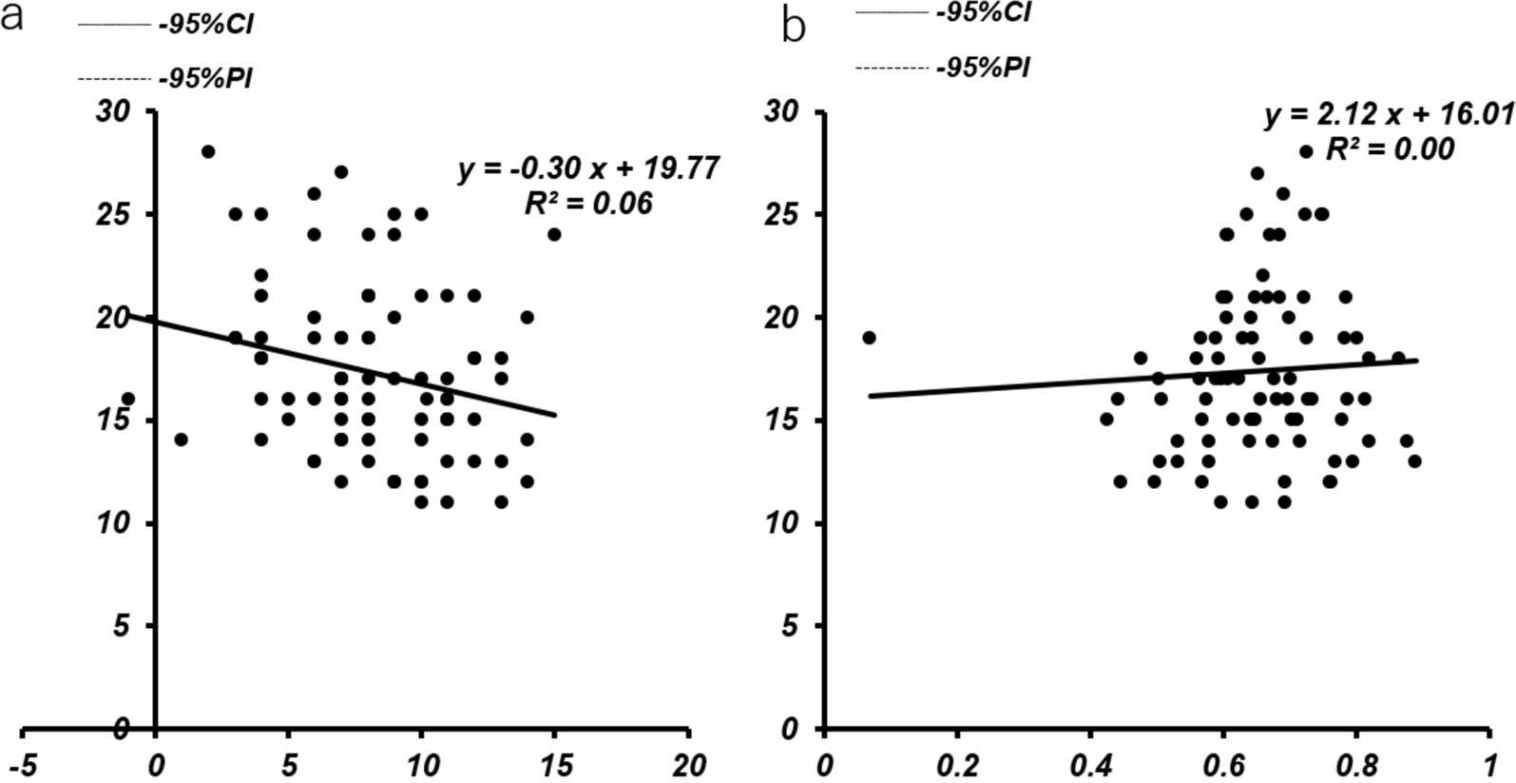
Correlation between the flexion gap without the trial component and posterior tibial slope angle (**a**) or patellar height (**b**)

**Fig. 6:**
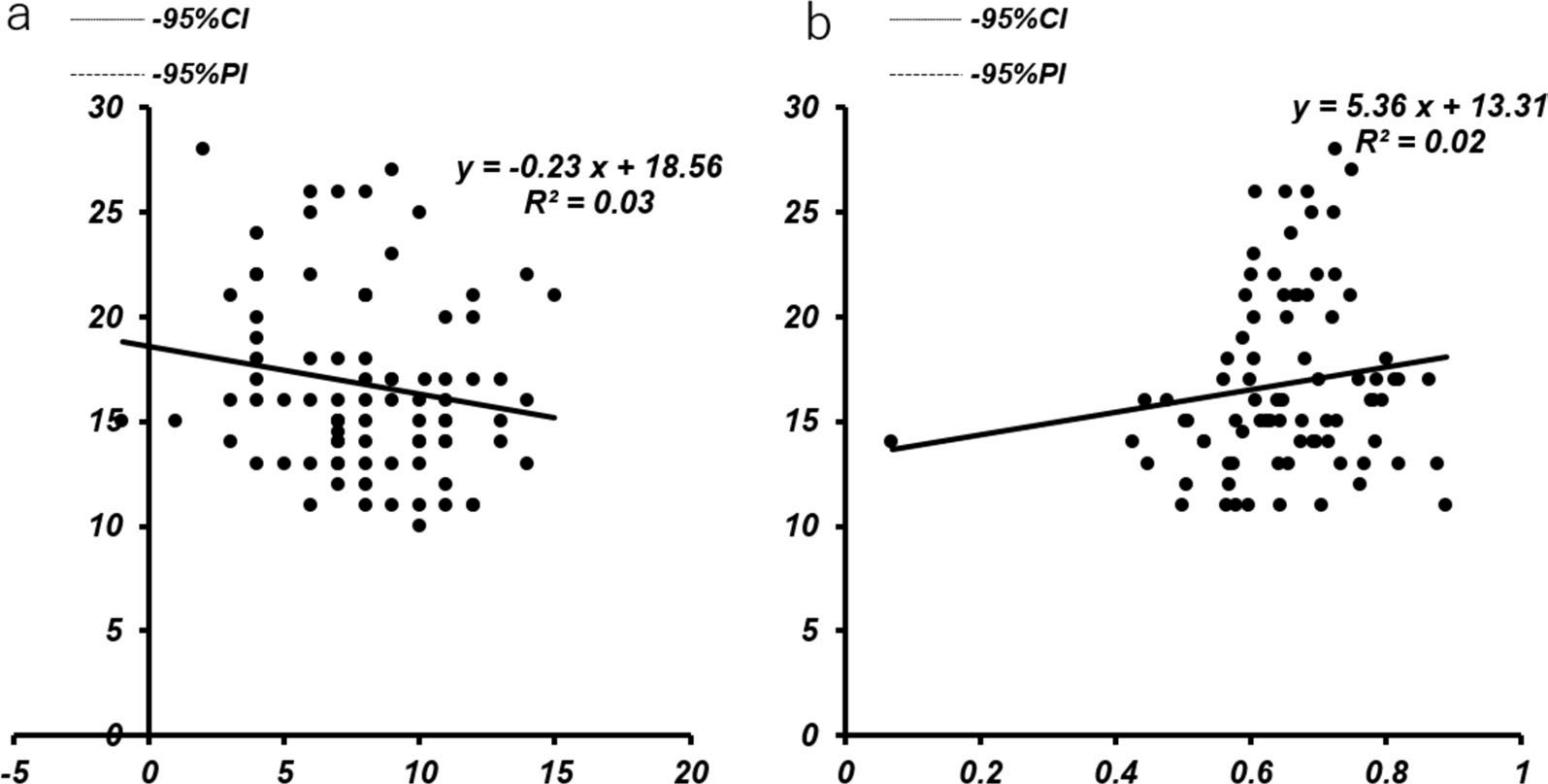
Correlation between the flexion gap with the trial component posterior tibial slope (**a**) or patellar height (**b**)

The flexion gap without the trial component was slightly correlated with posterior tibial slope angle (*r* = 0.238, *P* = 0.014) (Fig. 5a), and the flexion gap without the distal femoral trial component was not correlated with patellar height (*r* = 0.061, *P* = 0.289) (Fig. 5b). The flexion gap with the distal femoral trial component was not correlated with posterior tibial slope angle (*r* = 0.170, *P* = 0.060) (Fig. 6a) or patellar height (*r* = 0.149, *P* = 0.087) (Fig. 6b).

## Discussion

The key finding of this study is that the flexion gap size and inclination with the distal femoral trial was more reflective of the final femoral gap and inclination than the conventional bone gap (without the distal femoral trial). The clinical relevance of this study is that this method using distal femoral trial component will improve the accuracy of the modified gap technique TKA (Table 5).

**Table 5:** Comparison of gap size and inclination between with the distal femoral trial component and with the final femoral trial component

	With distal femoral trial component	Final femoral trial component	*P* value
Joint gap size (mm)	16.1 ± 0.46	16.1 ± 0.44	0.468
Joint gap inclination (°)	4.3 ± 0.46	4.6 ± 0.43	0.158

As flexion gap increases by cutting PCL, surgeons have been concerned about the flexion gap growing too large in PS-TKA [4]. Flexion gap has been reported to affect postoperative range of motion and clinical outcomes [13–15]. Therefore, adjusting the flexion gap is a crucial component of PS-TKA [16]. Other factors influencing the flexion gap other than PCL resection are patellar height, posterior tibial slope, and setting of femoral component. Some reports showed a correlation between flexion gap and posterior tibial slope or patellar height [2, 5, 6]: the higher the posterior tibial slope, the greater the flexion gap, and the greater the patellar height, the larger the flexion gap. These problems can be addressed by adjusting the line of distal femoral and proximal tibial cut and the tibial slope inclination. In this study, no significant correlations were observed between the flexion gap and distal femoral trial and patellar height/posterior tibial slope. Since the flexion gap tended to become larger as the tibial posterior slope increased, it will be necessary to perform osteotomies accurately using portable navigation and navigation to prevent the tibial posterior slope from increasing further.

Better postoperative results have been reported when the kinematics resemble those of a normal knee, and a soft tissue balance as close to that of a normal knee as possible is achieved [17]. With the use of navigation or robots, osteotomies and gap balancing have been becoming more accurate [18–20]; however, the soft tissue balance seems to be the most difficult to adjust owing to the degree of ligament release and individual differences; therefore, more detailed studies are warranted. Surgeons focus on the flexion gap when performing PS-TKA with the modified gap technique and understand the difficulties of adjusting the flexion gap balance. The rotation angle of the femoral component is an important factor affecting postoperative outcomes, including range of motion. In the modified gap technique TKA, it is important to measure the flexion gap as close as possible to the final femoral component setting after osteotomy of the distal femur and proximal tibia and determine the rotation angle of femoral rotation. The condition after final femoral component setting should be re-created using the distal femoral trial component when the flexion gap was measured in order to establish a precise soft tissue balance. It was reported previously that the joint gap size and medial tension were significantly reduced after setting the distal femoral trial component in CR TKA [10]. Since the modified gap technique has usually been performed for PS-TKA, it was essential to evaluate the effect of the distal femoral trial component with the PCL dissected. This study evaluated the effect of the distal femoral trial component on the flexion gap size and joint inclination, and the difference between those with and those without the distal femoral trial component in PS-TKA. Additionally, this study showed the extent to which the flexion gap size and inclination with or without the distal femoral trial component were reflected in the final gap size and inclination. The final gap size and inclination had similar results as the gap size and inclination with the distal femoral trial. Since results of the final gap size and inclination are more similar to those when using the distal femoral trial component, results using the distal femoral trial component should be used as a reference instead of the traditional bone gap (without the distal femoral trial) if the gap is set accurately.

There are three limitations to this study. First, this study was a retrospective study. Secondly, the rotation angle of femoral component was not determined according to the gap size and inclination with distal femoral trial component, and the intraoperative final gap and inclination were not compared with postoperative epicondylar view and postoperative clinical results. Finally, this study did not investigate correlation between modified gap technique using distal femoral trial component and clinical data.

## Conclusions

In the present study, the joint gap size and medial tension in PS-TKA were significantly reduced after setting the distal femoral trial component. The flexion gap measured using the distal femoral trial component was similar to that when the final trial component was set. To more accurately perform the gap technique TKA, the flexion gap should be measured using the distal femoral trial component.

## Data Availability

The data and material that support the findings of this study are available from the corresponding author.

## Abbreviations

TKA: Total knee arthroplasty
PCL: Posterior cruciate ligament
CR: Cruciate retaining
PS: Posterior stabilized
